# The impact of self-help groups on pastoral women’s empowerment and agency: A study in Nigeria

**DOI:** 10.1186/s13570-017-0101-5

**Published:** 2017-10-13

**Authors:** Adedamola F. Badejo, Ayodele O. Majekodunmi, Peter Kingsley, James Smith, Susan C. Welburn

**Affiliations:** 1 Centre of African Studies, School of Social and Political Science, College of Humanities and Social Science, The University of Edinburgh, 58 George Square, Edinburgh, EH8 9LD UK; 2 School of Biomedical Sciences, Edinburgh Medical School, College of Medicine and Veterinary Medicine, The University of Edinburgh, 1 George Square, Edinburgh, EH8 9JZ UK; 3 Livestock and Poultry Research Centre, University of Ghana, P.O. Box LG 25, Legon, Accra Ghana

**Keywords:** Women self-help groups, Zoonotic diseases, One Health, Livelihoods, Collective action, Pastoralism

## Abstract

While women in pastoralist communities are key stakeholders in the production of milk and dairy products for income generation, they are largely ignored in other areas of development such as health. The need to involve women self-help groups, in pastoralist areas in both animal health and human health development programmes, is essential, particularly given the high incidence of zoonotic diseases in these communities (Maudlin I, Eisler MC and Welburn SC, Philosophical Transactions of the Royal Society B: Biological Sciences, 364(1530):2777-2787, 2009). Understanding the process and impact of social networks on livelihoods is essential for any development programme that aims to prevent and control zoonotic diseases. This study examines the roles and responsibilities of women self-help groups in Kachia Grazing Reserve and Bokkos, Jos Plateau, Nigeria.

The findings show that groups promoting social, physical and psychological health strongly motivated women’s involvement in self-help groups. Self-help activities showed commitment to effect a change in their livelihoods, despite constraining environmental, cultural and social factors. Engagement of women’s self-help groups in livestock development programmes offers a powerful instrument for driving forward the One Health practice in pastoralist communities, promoting human, animal and environmental health and well-being.

## Introduction

Collective action (CA) is defined as the act of mobilizing people around common concerns to harness the ‘power of the group’ to solve their problems. In terms of agency, collective action specifically seeks to help diverse groups of poor and socially excluded citizens, including women, to organize themselves, exercise voice and choice and demand broader institutional change to improve their lives and livelihoods. It is a route to local problem-solving and increased social accountability, able to overcome cultural, political and institutional barriers to improve people’s lives (Mahmud 2002, Evans and Nambiar 2013). Discrimination against women remains a global issue. Pastoral women bear a particularly heavy burden in terms of discrimination, being doubly marginalized - first as pastoralists who live at the margins of national economic and political life, secondly as women living within the patriarchal cultural contexts of gender inequalities and injustices. There is a long history of collective action by women’s groups which has been a potent force for women’s empowerment that has inspired, initiated and facilitated women to highlight and solve shared problems in areas including economic empowerment, gender-based violence (GBV), child marriage and ethnic and religious violence. A notable example is the Pastoral Women’s Council which facilitates women’s collective action to tackle women’s economic empowerment, education and leadership for Maasai women in Tanzania (Ngoitiko 2008).

The economic theory of collective action is based on the concept of provision of public goods through collaboration. In poor, rural areas, grassroots collective action groups are an effective response to state and market dysfunction and weak or missing state institutions. This is particularly important for groups with limited access to public goods where available such as women, the poor, ethnic minorities, scheduled castes and tribes (Mansuri and Rao 2012, Ostrom 2015). An alternative view questions the effectiveness of the bottom-up approach of CA and emphasizes linking top-down government action with local CA problem-solving to resolve constraints to the state delivery of public goods (Booth 2012, Agarwal 2000). Both approaches see CA as a way to solve the problems of weak/missing state action to improve outcomes for citizens. There are three interlocking *instrumental* aspects of CA which help women advance specific goals: actions to solve public-goods problems that directly impact women’s lives, assets and livelihoods; actions that expand opportunities for women to exercise voice, influence and agency and enhance their decision-making power; and actions that explicitly challenge social norms and behaviours that constrain women’s agency in household and public domains, irrespective of their social status (DFID-Nepal 2010, Meier zu Selhausen 2012, Pandolfelli et al. 2008).

Collective action also has an *intrinsic* value aspect which is the link between the ‘act of associating’ and women’s psycho-social well-being. The act of association becomes a resource, vital in developing self-confidence and self-esteem by providing spaces and networks for women beyond family and kin. Developing this ‘power within’ helps women go on to challenge gender norms in society as individuals and in CA groups by establishing positive feedback loops between self-worth and agency. The absence of CA is a key contributor to experiences of disempowerment for both men and women (Evans and Nambiar 2013). Both the instrumental and intrinsic aspects of CA are essential to its transformative power for women and society in general. However, change that does not occur in all three instrumental domains will not be transformative (Evans and Nambiar 2013, Kabeer 2012).

Not all collective action is positive. CA efforts can be used to oppose development reforms or to project the interests of one group to the detriment of others (Corduneanu-Huci et al. 2012). Similarly, not all collective action is successful. Sustaining a group is a continuous process of contestation and negotiation. External factors that determine the success of CA are very context-specific, depending on local institutions and incentives, pre-existing levels of social cohesion and inequality and prior experiences of CA. Links between CA and women’s agency are clearly complex and context-specific. There is no single, linear pathway linking CA and improvements in women’s status. Instead, a series of reinforcing actions is usually necessary to ensure positive impact and transformative change.

There is a strong pastoralist presence in the sub-humid zone of Nigeria - a very diverse region with multiple cultures, religions and lifestyles living side by side. Most Fulani pastoralists live in mixed villages alongside ‘indigenous’ farmers, but some reside in grazing reserves. The positive and negative impacts of sedenterization on pastoral women’s lives are well documented (Hannah 2007, Flintan 2008, Fratkin and Roth 2006), but less research has been focused on the impacts of where pastoralists settle and who they live amongst. Such information is key in view of the recent escalation of farmer-herder conflicts in Nigeria and the ongoing national debate on grazing reserves (Majekodunmi 2017).

This study considers two different types of Fulani settlements in Nigeria: the Kachia Grazing Reserve (KGR), a gazetted pastoralist enclave in Kaduna State, and pastoralists settled within ethnic Ron villages in the neighbouring Plateau State. The study describes the forms of women’s CA exhibited within these communities and the social and gendered normative contexts in which they exist. It also examines the impact of CA on expressions of women’s agency such as access and control of resources, decision-making in household and public domains, political participation and societal influence.

## Study area


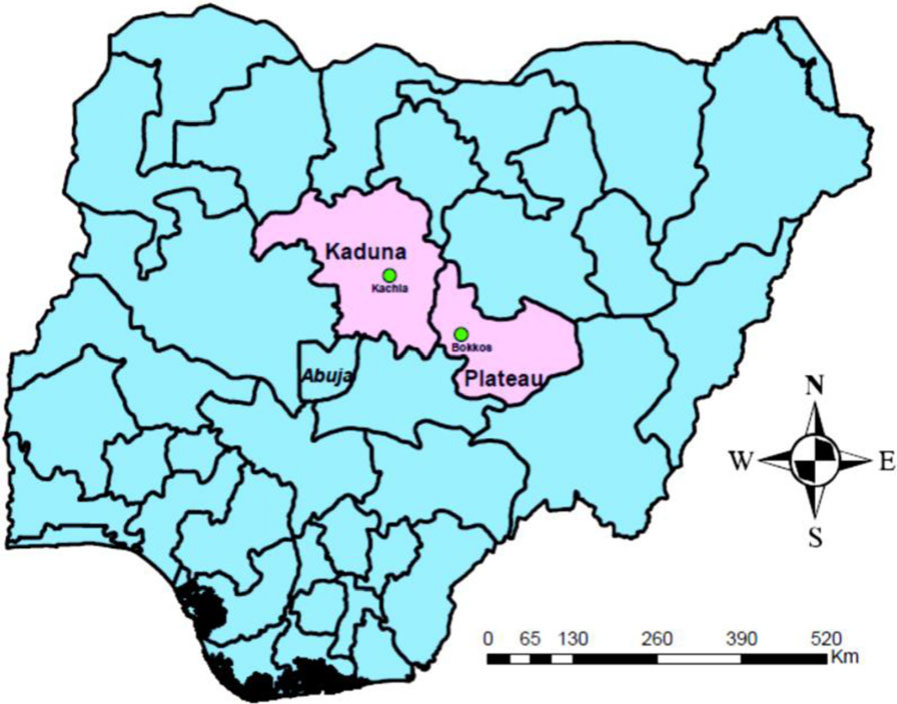


### Kachia Grazing Reserve

Kachia Grazing Reserve is situated in Kaduna State in northern Nigeria. It has an area of 334 km^2^ divided into six administrative blocks with distinct demographic and ecological characteristics. The reserve is populated exclusively by ‘settled’ Fulani pastoralists practicing seasonal transhumance. The quality of soil and pasture in KGR are poor (Ducrotoy et al. 2017a). While the area was declared free of tsetse flies after a successful spray programme in 1967 (Oxby 1984), subsequently, tsetse flies have reinvaded; this and poor pasture remain a serious problem for animal health on the reserve (Majekodunmi et al. 2013). When first established, KGR was well equipped with infrastructure such as veterinary and health services, dams, boreholes and fodder banks by ILCA (Kaufmann et al. 1986; Oxby 1984). Most of these services are no longer provided, and physical infrastructure within the reserve is in poor condition. The reserve is isolated with only a single dirt road providing access.

The KGR is an agro-pastoral mixed herding and cultivation system. Sources of income include sales of livestock, dairy products, crops and off-farm activities. Around 90% of households in the KGR grow crops, mostly for subsistence (Ducrotoy et al. 2016, Ducrotoy et al. 2017b). Livelihoods are characterized by high cash needs, high sale rates and low prices for cattle linked to the isolation of the Grazing reserve. Cattle productivity is just high enough to support Fulani households without depleting herds. The limited pasture within the reserve and continuing political insecurity in surrounding areas are pushing KGR pastoralists into poverty, forcing them to keep smaller herds and diversify their sources of income (Ducrotoy et al. 2016, Ducrotoy et al. 2017b).

There is marked socio-economic stratification within the reserve; wealthier, long-established settlers reside near the central market with access to services while more recent and poorer settlers live farther away with less favourable access to all amenities. KGR is a predominantly closed community with strong customary authorities and scant engagement with government. It is considered a safe haven for Fulani fleeing violence and insecurity elsewhere and experiences significant immigration of internally displaced persons (Ducrotoy et al. 2017a, b).

### Jos Plateau

The Jos Plateau in North-Central Nigeria covers an area of 8,000 km^2^ at an altitude of 900 to 1700 m. The Plateau is comprised of granite of volcanic origin, and the terrain is characterized by numerous rocky, flat-topped hills and crater lakes. Vegetation, formerly open savannah woodland, is now mostly grassland. The Plateau is a highly populated, intensively cultivated area with intense animal production that plays a significant role in national cattle production. The Plateau was traditionally considered attractive for pastoralist settlement due to being free of tsetse flies and African animal trypanosomiasis (AAT), although there have been confirmed reports of tsetse and AAT on the Jos Plateau since 1982 (Majekodunmi et al. 2013a).

The Plateau is inhabited by a great variety of indigenous ethnic groups with complex clan organization and ritual kingship systems and correspondingly shows high levels of ethno-linguistic diversity. Jos city is cosmopolitan, with inhabitants from across Nigeria and a significant Muslim/Hausa presence (Fricke 1979; Awogbade 1983; Blench 2004). Rural areas are dominated by farmers of Plateau tribes with a significant minority of settled Fulani herders.

Fulani settled within the Bokkos local government area (LGA) are transhumant agro-pastoralists. Livestock sales provide their main source of income, along with crops for sale and subsistence and diverse off-farm activities. Despite the challenges related to insecurity and limited access to natural resources, breeding herds are large and show above average productivity and reproductive performance (Majekodunmi et al. 2016). High natural herd growth and moderate offtake allows households to maintain and increase their herd sizes. Increased transhumance and use of hired herders has allowed them to mitigate the risks of natural resource conflict and insecurity but the wage bill is high. Plateau villages are connected by a good road network and have high proximity to markets, which results in high cattle prices.

Fulani on the Plateau are subject to customary authorities in their ‘host’ villages, with their own *Ardos* working under village chiefs; the elected *Ardos* may be deposed in cases of grave misconduct. The importance of customary authorities, however, is in decline, while levels of influence and engagement with government and political authorities are increasing (Majekodunmi 2017).

The Ron (Challa) people of Bokkos LGA are traditionally farmers and hunters. The area has very fertile soils and is the centre of potato production in Nigeria. It is a patrilineal society organized by exogamous clans and by membership of associations which controlled political, religious and judicial matters. Separate men’s and women’s associations exercise considerable authority, with men’s associations being the more powerful. The Ron practice Christianity, although some still observe their traditional religion.

## Methods

In-depth interviews were conducted at the household level with selected Fulani and Ron households in six villages in Bokkos LGA, Plateau State. Six Fulani and six Ron households were selected in each of the six study villages. Village and household selection were purposive as a consequence of volatile security situation. Subjects explored during interviews included nature and membership of collective action groups, women’s economic activities, influence and agency and gender relations.

Within the KGR, 21 self-help groups (both male and female) were identified of which six were women’s self-help groups. Two focus group discussions (*n* = 6 members for each FGD) were held with the representatives of all six women’s self-help groups in the reserve. Additional focus group discussions were held with village authorities in each of the six administrative blocks in KGR. A facilitator/translator fluent in Hausa and Fulfulde (the two languages spoken within the community) assisted the author in conducting the focus group discussions. Two age groups, a younger age group (between 15 and 35 years of age) and older age group (between 36 and 65 years of age) made up the focus groups. Participants were specifically asked about group organization, their activities and any constraints. Questions were also asked concerning women’s economic activities, contributions to livelihoods, influence and agency.

Qualitative data was entered into Microsoft Word and analysed manually based on accepted methods of coding and memo-writing (Bourgeault et al. 2010; Padgett 2011). Data was subject to thematic qualitative analysis - coding into themes of women’s mobility, income-generating activities, decision-making, influence, position, types and impact of women’s collective action.

## Results

### Collective action groups

#### Fulani in Bokkos, Jos Plateau

No active women’s self-help groups were identified amongst the Fulani women in the Plateau study area. One respondent mentioned that one such group called *Sumpo* (relationship) had been formed two years ago but was no longer active. Its primary function was to discuss Fulani women’s welfare concerns.

#### Kachia Grazing Reserve

Six functioning women’s self-help groups (SHGs) were identified in KGR: *Wuro Nyako* (House of Nyako), *Wuro Fulbe* (House of Fulani), *Mayo Borno* (River Borno), *Wuro Lobi* (House of Lobi), *Wuro Tale* (House of Tale) and *Habbanaye* (We have tied one). All six groups come under an umbrella organisation *Bige Weti* (We are enlightened) which coordinates their activities. The criterion for membership is marriage:

‘Single ladies look forward to being married so they can become members of any group of their choice, as being a group member is a sign of prestige and increased social status’.

The motivation of the women for joining SHG groups included a desire to earn more income so as to fulfill their financial responsibilities in the household; this included education, especially for girls. They expressed a desire for their children to be educated and appreciated that the only way to ensure this is to pay for it.

‘We women, we are the ones paying the school fees for our children and most of the salaries of teachers from this school are paid for with our money from this society. If you want your children to go to school, you have to pay’.

‘We are the ones responsible for the payments of fees because the men are not willing or ready to pay for the education of our daughters’.

Each SHG had its own administrative committee, composed of a leader who must be educated at least to the primary school level, a secretary, a treasurer and two or three other members. Committee members are mostly in the 15- to 35-year age group. However, neither *Bige Weti* nor any of its constituent groups were registered with the government. Mentoring young brides on the expected roles of a wife within the household was an important activity in these groups.

‘Because the majority of us marry at a young and are naive about some important women’s issues and the intricacies of household maintenance, we carry out activities aimed at building and strengthening these young women’s conjugal relationships’.

The younger women admitted that the tutelage of the older and more experienced married women helped to build both their knowledge and confidence in meeting the needs of their husbands and families.

‘We desire to please our husbands and maintain the household, that’s why we are involved in many of the groups’ economic and social activities (Female FGD participant 15 to 35 years)’.

Services to enhance members’ income-generating activities are commonly undertaken by the SHGs. Some SHG activities were funded by external NGOs, facilitated by personal connections of SHG members rather than formal partnerships. These organizations donated small ruminants, sewing machines and funds and resource persons for training. The SHG also provide savings and loan facilities to members. Members make weekly contributions into a common pool, and from this, members can access loans for their businesses.

Livestock management is another SHG activity to promote economic gain for women. Sheep purchased by or donated to the group are distributed to the members to rear for a fixed period. Any lambs born to the sheep become the property of the member, while the original sheep remains the property of the group. This serves as an incentive for commitment and hard work and allows women to build up their own sheep herds. It also means that the group always has sheep available for rotation amongst members.

Sanctions are applied against defaulters to ensure regular contributions and repayments are made in the form of a delay in granting of a loan or withdrawal of SHG sheep from defaulters.

SHGs also organize skills acquisition training for women in cottage industries, e.g. the manufacture of soap and moisturising cream, snacks such as groundnut cake and chin-chin and agro-forestry products such as locust bean condiments and ‘cocoa’ drinks from baobab seeds.

NGOs seeking formal partnerships found it difficult to work directly with women because their SHG were not registered. Women’s SHGs were only able to participate in a formal partnership with an NGO in collaboration with the registered men groups during an HIV/AIDS campaign. As a result of the non-registration of women groups and non-collaboration with other agencies, the women’s groups have missed many opportunities that could have helped them accomplish many tasks significant to the agency of women.

‘NGOs prefer to partner with the men’s groups because they are well grounded, stronger and more influential’.

The women groups also play a significant role in the educational system of the KGR. The primary school used as the FGD venue was built by the women’s SHGs who also paid teachers’ salaries. However, this has now been taken over by male village authorities.

Administrative blocks 1 and 2A of KGR were said to be inhabited by the elites of KGR society, the wealthiest families, and the earliest settlers in KGR. These blocks comprise the ‘urban centre’ for life in KGR, housing most of the amenities, including the road, market, health centres, schools, dams and bore holes (Ducrotoy et al. 2017b). The poorest families reside in blocks 2B and 6, which have poorer soil and are farthest from any amenities. Inhabitants of block 6 are considered the lowest ranking members of KGR society, socially disadvantaged by their isolated location and ‘slave’ status. The ‘slave’ women from block 6 did not participate in any of the women’s SHGs.

#### Ron indigenes, Bokkos, Jos Plateau

Four types of SHGs were recorded amongst the Ron women: *Kungiya adache mata* - women’s thrift groups, *Kungiya Zumunta Mata* women’s (church) fellowship groups, NGO-organized groups and ‘committee of friends’ fund-raising and support groups.

The *Kungiya adache mata* thrift groups are informal weekly contribution groups run as described above for KGR.

The ‘committee of friends’ are short-term ad hoc groups formed to support a friend or relative planning a social occasion, e.g. a wedding, naming ceremony and birthday party. They may be men’s, women’s or mixed groups. Committees of friends contribute cash and required items (food, drinks, material, etc.), undertake fund-raising activities and assume responsibility for planning and organizing the event.

*Zumunta Mata* (women’s fellowship) groups began as a grassroots movement in protestant churches across northern Nigeria in the late 1960s. *Zumunta Mata* was part of a wave of Christian expansionism sweeping northern Nigeria at the time along with the *Sabon Rai* (New Life) movement that were embraced by the Catholic church in the mid-1970s. *Zumunta Mata* is found across all established Christian denominations in Northern Nigeria today; ‘To be a Christian woman in the north is to be a member of Zumunta Mata’ (Enwerem 1995). *Zumunta Mata* has great influence in the economic, social, political and religious lives of northern Christian women, with ecumenical impact on the church, breaching the barriers between Christian denominations and enriching the litany through inclusion of traditional music and instruments. These groups constitute a bona fide theological community with significant contributions to evangelism, promoting Christian living and inspiring religious vocation (Ritchie 2001).

In terms of agency, *Zumunta Mata* has redefined women’s roles and given them a strong voice and platform within society. *Zumunta Mata* is ‘very powerful’, able to exert considerable influence to gain security for women, widows and girls through effective engagement with traditional, administrative and political authorities. *Zumunta Mata* has successfully reduced the impact of many negative cultural practices. Men acknowledge the power of this organization and engage with it in various ways, some leveraging the influence of the organisation to mediate with women as a group in society or in individual marital disputes. Others see *Zumunta Mata* as a threat and seek to limit its influence by forbidding their wives to join (Ritchie 2001, Shebi 1997, Para-Mallam 2012).

Some respondents reported membership of NGO-organized groups, including the Country Women’s Association of Nigeria (COWAN). COWAN was founded in 1982 to tackle poverty and marginalization of rural women (Salau et al. 2012, Ogunleye 2006) and is active in 32 of the 36 states in Nigeria, with over 260,000 members and 9,000 registered community groups. COWAN provides microfinance, socio-political awareness and healthcare services to members and offers vocational-training, leadership-training and training in adult literacy. COWAN mobilizes and organizes rural women into SHGs and registers these groups under its ‘franchise’. SHG activities include microfinance savings and loans in which women make weekly savings and are eligible for loans of twice the amount saved (Ogunleye 2006, Salau et al. 2012).

Health-related activities include community health-screening days, training and placement of community health workers, establishment of women and youth friendly clinics in communities, family-planning services and awareness events on HIV, cancer, nutrition, etc. COWAN provides emergency health insurance scheme for members and immediate family members (N20 ($0.06) weekly subscription), which funds many caesarean sections and other maternal health procedures.

Socio-political activities include awareness talks which have served to reduce the vulnerability/susceptibility of rural women to unscrupulous ‘vote-buying’ electoral campaigns. COWAN also supports electoral campaigns for female candidates for political office with a record of peaceful, orderly campaigns with 80% of funded candidates elected. Women members contribute N10 ($0.03) per month to this fund and approximately 5 million naira ($13,886) is disbursed annually to cover registration fees, flyers, banners, etc.

The Plateau State chapter of COWAN partners with national and international agencies on many activities including agricultural training and extension activities for vegetables, violence against women and girls, and HIV awareness (COWAN 2017).

Members of COWAN are either married women or widows; most are Christians although COWAN is non-religious and non-denominational. Lower participation by Muslim women may be due to specific barriers in their participation, or they may feel out of place and unable to relate to other members, if COWAN is perceived as a ‘Christian’ organisation.

In conclusion, the Ron women’s groups include both formal and informal groups with local, national and international reach. Most of these groups are registered with the government. They play an important role in women’s lives and in the wider community giving women influence at the household, church, and community levels.

### Social capital, collective action and agency

#### Fulani in Bokkos, Jos Plateau

Fulani women are isolated and feel their isolation. Although they are not officially in purdah/seclusion, they are not permitted to attend the mosque or participate in celebrations such as weddings and naming ceremonies within the Fulani community (despite preparing and cooking the food for guests). Some are not permitted to go to the market, and in these cases, the husbands or elderly women (past menopause) within the household will buy all foodstuffs. Most women regarded their limited freedom of movement and association as discrimination, but a few did not - their argument was that any practices in line with Islamic observance could not be considered discriminatory. Most women desired greater freedom of movement to improve their social lives.

‘We are denied the right to socialise’.

‘No free movement and not allowed to associate freely’.

Decision-making and influence of the Plateau Fulani women was limited to that within the household. Most women interviewed were involved in decision-making within the household but not within their community. Decision-making at the household level was limited by age, with only older women being consulted. Women have no say in the bargaining process for the dowry of their daughters nor in the management or sale of their own cattle.

Income-generating activities for women were limited to small-scale trade in dairy products, poultry, small ruminants and in condiments - activities that were carried out at home. Some had their own market gardens for generation of ‘cash crops’ (e.g. cabbage, lettuce, carrots, green peppers). Dairy sales were seasonal, mostly within the wet season and mostly sold to wholesale customers who picked up the dairy products from the homestead. A few (mostly older women) sold their dairy products at the market. In this community, 12% of households did not have milk income during the wet season and 24% during the dry season (Majekodunmi et al. 2017). A third of the respondents reported not having any income-generating activities. One respondent mentioned that some men viewed women’s trade in livestock as competition:

‘Men often say that women keeping animals are competing with them’.

Any women’s generated income was spent on supporting the household, e.g. on soap, cooking oil, condiments and children’s clothes. Despite only 34% of households being entirely dependent on the male household head (Majekodunmi et al. 2017), the women’s financial household contributions were limited - the burden for providing fell mostly with the husband. Most women considered their own financial contributions to the household insignificant. Only two respondents considered their contributions significant. One respondent had multiple sources of income (selling sweaters made on her knitting machine, dairy products, poultry and small ruminants) and made more significant financial contributions to her household. Both men and women felt that women’s most important contribution was to cook and raise the children. Men did acknowledge the roles/contribution made by women in these areas.

‘Source of income is not that important as our men provide everything’.

‘We sell nunu (local milk) during period of abundance but when not available we fold our arms because our men provide everything’.

‘Our role is to cook and bring up the children’.

The women did acknowledge that their income was not dependable.

‘Income from the sale of poultry and small ruminants bring in income as ‘nunu’ is no longer dependable’.

Some desired better opportunities for income-generating activities to earn income and simply for something to do, and some admitted that their needs were not always met by their husbands.

‘Sometimes our needs are not met’.

‘Husbands do not always meet up with their financial obligations’.

#### Fulani in Kachia Grazing Reserve

Fulani women in KGR had a wide variety of income-generating activities. They sold agricultural outputs (dairy products (limited) and farm produce), agro-forestry products (fermented locust bean condiments and baobab drinks), handicrafts (beads, mats and calabashes), home-made soap and moisturising cream, snacks, beverages and condiments; owned businesses such as canteens, seamstresses and commercial grinding machines; and earned wages as teachers, nurses and cleaners in local schools and hospitals within the reserve.

Women had freedom of movement within and outside the reserve. Many travelled outside the reserve periodically to buy goods despite the bad road and high cost of transportation. Both men and women see women’s roles as equal contributors to household finances and community development. The women also played a significant role in the education of their children. Proceeds from their economic activities went to the purchase of school uniforms, stationery and school fees - especially for girls. Men appreciated women’s efforts in these areas:

‘If not because of women in this community we would not have developed like this. Women are trying in this village. Whenever the males go to their various place of work, so also the women go, cook for their children and send them to school, so women are really trying’.

Women in KGR did not attend the mosque. They participated in decision-making at the household level individually and at the community level only through their SHGs. SHG agency within the community was significant but limited to women’s own affairs, rather than community-wide issues. They had little interaction with customary or state authorities except to receive orders from customary hierarchy (Okello 2013).

SHGs provided opportunities to socialize, air views and challenges and get help and were considered a valuable support network. Women could join more than one group. SHGs within KGR were, however, not available to the poorest inhabitants of block 6 who were marginalized due to their ‘slave’ status and not allowed to join SHGs.

The SHG system in the Grazing reserve is seen to have enhanced group identification, cooperative spirit, self-confidence, interpersonal relationship and confidence in the group members with the exception of the poorest in the community.

#### Ron indigenes in Bokkos, Jos Plateau

Indigene Ron women reported being engaged in a wide range of economic activities including crop farming, animal husbandry (cattle, sheep, goats, pigs, poultry), processing of agricultural products, wholesale and retail agricultural trade in agricultural products, purchasing of farmer produce to sell on market days, catering services, seamstress and trading in goods and services. Some were employed as nurses, school teachers, civil servants, etc. Many owned their own farms, separate from the family farm(s) owned by their husbands. These were either bought with their own money or given to them by their husbands. Some owned cattle.

Ron women reported full participation in community affairs with the exception of politics where they felt marginalized. Indigene Ron women considered themselves essential to the household economy and in some cases more so than men. They considered their roles and contributions to the family, church and community as *vital* and *irreplaceable*.

Women reported contributing to all household expenses alongside men. Some making monthly contributions to the household ‘kitty’. The male perspective was that, ‘Most of us that marry more than one wife care little about household needs’*.*

Their membership of various groups, societies and associations were considered an important social and emotional outlet, providing space and opportunities to socialize. Women reported playing an important role in church and view full participation in church activities as both a personal religious duty.

‘We play a vital social and economic role in the household and community’.

‘We determine the status of the household’.

## Discussion

The general trend of Islamic reform in Nigeria and the Izala movement, in particular, is exhibiting a visible impact on Fulani communities on the Jos Plateau. *Jama’atu Izalatil Bid’a Wa’ikamatis Sunnah* (JIBWIS) translated as the Islamic Organisation for Eradicating Innovation and Establishing Sunnah, the dominant religious movement amongst the Plateau Fulani, was founded in Jos in 1978 as an anti-Sufi movement with an agenda of Islamic reform and orthodoxy as well as independence and self-reliance (Higazi 2013, Ostien 2012, Hickey and Thompson 1981). Under Izala, the position of women in Fulani society has been significantly compromised impacting on their social and economic status. Firstly, Izala has introduced greater restrictions on the mobility of women; Fulani women are mostly confined to their household compound and rarely leave their community. The economic activities with which they are able to engage have been restricted, with Fulani women being discouraged from their traditional practice of selling their dairy products in public and attending local markets (Higazi 2013, Ostien 2012). Secondly, women are positively excluded from participation in the JIBWIS administration and active membership of its aid group - a position unique amongst all the Islamic groups in the Plateau State (Moddibbo 2012). Thirdly, dealings between Fulani and indigenes have been restricted, with interactions mostly confined to public spaces. These groups no longer visit each other’s homes or exchange food/gifts at Christmas or Eid. Recurring violence on the Plateau since 2001 has served to reinforce this pattern of community isolation (International-Crisis-Group 2012, Taft and Haken 2015). The combination of these factors has served to limit women’s agency, bargaining power and capacity to spontaneously self-organize (Desai and Joshi 2013, Evans and Nambiar 2013).

Fulani women residing in Bokkos on the Jos Plateau, did not have any women’s self-help groups. In general, they had only limited mobility and few opportunities to socialize or participate in religious or income-generating activities. The main reasons for the restricted mobility of women on the Jos Plateau are the dominant Islamic sect in this area and the pluralist nature of rural villages on the Plateau. Access to SHGs could meet the Fulani women’s expressed needs for social interaction and increased income-generating activities.

The cultural and religious factors observed on the Plateau that limit women’s mobility and economic activities have had less influence amongst Fulani in the KGR. The KGR is a closed, homogenous community setting with a strong top-down authority structure that permits little state interference. It is a less prosperous area with livelihoods beset by low productivity of land and livestock and expensive goods and services. KGR Fulani can afford to ‘relax the rules’ to improve their livelihoods for economic gain since there is less threat of non-conformity.

The Jos Plateau, in contrast, is a mixed society divided along ethnic and religious lines - here the Fulani are an ethnic minority, experiencing segregation and self-imposed separation from their ‘indigene’ neighbours. Bokkos Fulani show strong adherence to cultural norms to preserve their way of life in the face of their minority status and ample opportunities for non-conformity (Berry et al. 1989, Atkinson et al. 1998, Cormack 2016). Governance is civil rather than cultural with considerable state engagement. Plateau pastoralists are relatively prosperous, with opportunities for children’s education and for diversification of livelihoods for the men, and there is less ‘need’ for additional income generated by women in the household. Some men within the community view women’s income-generating activities negatively and as ‘competition’. In contrast, KGR and Ron men see women’s efforts and economic contributions as legitimate and acceptable.

Fulani women’s groups in KGR provide a safe space for social interaction and a valuable support network for counselling, mentoring and various income-generating and community development activities. The major goals of Fulani women in KGR are to successfully grow into their roles as wives and mothers and improve their own income and assets, their household standard of living and the education of their children. Nonetheless, the membership, activities and impact of these SHGs in KGR are compromised in several ways. Firstly, their scope is restricted to the KGR and the absence of registration of these groups means that they cannot collaborate with organizations that do have the capacity to spread their scope beyond KGR. Secondly, women of lower socio-economic status are excluded from membership. Thirdly, they operate firmly within prevailing social and gendered norms. That they do not seek to change the prevailing social and gendered norms restricts their influence and impact to traditional ‘women’s activities’ that are not allowed to spill over into traditionally male-dominated areas. An example of this was the appropriation by male village authorities of the school built and established by the women’s SHGs. When women’s SHGs operate within pre-existing hierarchies and negative gendered social norms, the status quo of disempowerment is maintained and this serves to reduce the positive impacts of women’s collective action (Westermann et al. 2005).

The scale and duration of CA benefits depend on how explicitly programmes seek to shift social norms. CA will not enhance agency without challenging both formal and informal normative frameworks. This is a prerequisite for transformative agency. When this is not a stated goal, then benefits form one domain will not spill over into others and empowerment impact is likely to be low and partial, rather than transformative. This is a common feature of collectives focused only on economic outcomes which achieve increased income and control over resources for women but have no positive impact on health, household decision-making, independence, Gender Based Violence (GBV) or gendered social norms (Baden 2013, Beath et al. 2013). The SHGs within KGR do not seek to challenge social and gender norms, and therefore, their impact is limited rather than transformative. Despite these limitations, women in KGR derive intrinsic and instrumental benefits from their SHGs. An increased sense of self and of self-esteem reinforces the gains in influence and economic empowerment, and over time, SHGs can erode restrictive social norms and increase women’s agency (Sharma and Sudarshan 2010).

Where existing levels of social stratification are replicated within CA groups, the impact on escape from poverty and social mobility are weak, whereas CA groups that challenge existing social stratification hierarchies lead to transformative change (Narayan et al. 2009). In northern Kenya where membership of women’s CA groups focused on the poorest members, there was high level of poverty escape and social mobility (Coppock and Desta 2013, Coppock et al. 2006). Mahmud (2002), however, found that for groups in Bangladesh that did not actively challenge social hierarchies, motivation for participation and benefits were higher for the elites and the overall effect of CA on poor women was negative, since they were crowded out of the groups and pushed further into poverty.

Significant class/social stratification was observed in KGR society, and these are replicated in the women’s groups. Leadership positions are reserved for literate women and tended to be given to women of higher status in the community. Women residing in the poorest administrative block of KGR, block 6, were entirely excluded, based on their class and social position. Since they are also the poorest and most socially excluded in the community, they did not derive any of the benefits from the SHGs (intrinsic benefits of association and instrumental impacts on income, education and agency), and SHGs in KGR had no impact on social mobility.

Amongst Ron indigene women, there were several types of women’s SHGs, both formal and informal, that exhibited scope at the community, state, national and international levels. *Kungiya Zumunta Mata* and COWAN specifically sought to challenge negative social and gender norms. These groups showed positive effects on women’s agency in all spheres of life and successfully reversed or minimized negative social and gender norms in addition to providing a vibrant platform for women’s interaction and influence with state and customary authorities. Such groups have positive effects on individual economic outcomes for group members, including higher income, productivity and product quality and better access to credit, market information, training and technology. They also have widespread benefits for all women in the community - increased mobility, access to employment control of household expenditure, political participation decision-making and bargaining power at home and workplace/market (Desai and Joshi 2013, Deininger and Liu 2009).

Restrictive membership requirements are known to impact on women’s participation in collective action. ‘If you are married people respect you; but if you are “roaming about” unmarried you don’t get respect. A woman who is not married is still a “girl” and is not respected. Even if you are a girl (age-wise) if you are in *gidan aure* (the house of marriage) you will be given respect’ (Para-Mallam 2012).

Amongst both KGR Fulani and Ron women, marriage is a criterion for group membership and unmarried girls and women are excluded.

Demographic change and rising unemployment in Nigeria mean that improving economic opportunities for the youth is a top priority for economic and social development. This is particularly important for young girls, as employment serves to reduce risks of future dependence on men and promotes agency and empowerment. Interventions for avoidance of early marriage, sexual and reproductive health, education and financial literacy, provision of safe spaces and peer support for girls have more impact when girls are engaged in groups. Community-based groups for adolescent girls deliver both instrumental and intrinsic benefits of collective action (Evans and Nambiar 2013).

Religious observance and women’s religious groups play a key role in women’s empowerment amongst the Ron. These aspects were largely absent in both groups of Fulani women in KGR and on the Plateau. This is primarily because these Fulani women do not attend prayers at the mosque and are therefore excluded from organized religious establishment and the world of women’s Islamic groups. These differences have been previously observed in these communities; Para-Mallam (2012) observed that ‘Religion (Islam) and age-long traditional prejudices are responsible for (Hausa-Fulani) women’s subjugation. In contrast, it is religion (Christianity) through *kungiyar zumunta mata* that provides space for considerable female influence’. Women’s mosque attendance has been a contested issue since the death of the Prophet Muhammad. There was an early consensus against women’s mosque attendance; however, this has not been constant or universal, with plenty of variation in time and space. The decline in women’s mosque attendance is evidence of the progressive erosion of their status in Islamic society after Prophet Muhammad’s death. It is also linked to ascendancy of the concept of *fitnah* (temptation) which argues for greater separation of sexes.

Globally, there has been resurgence of women’s mosque-based activities since the 1970s. Mosque attendance and membership of women’s Islamic groups are acknowledged to empower women; women become visible in public and religious arenas, and they gain more influence and social capital by becoming more ‘religious’ and are better able to negotiate their gendered position within social and institutional arenas (Nageeb 2007, Badru and Sackey 2013, Katz 2014, Obadare 2016). Islamic women’s groups exist across Nigeria. They play a particularly prominent role in religious life amongst the Yoruba in the South-West - in line with Yoruba culture in which women’s groups have always played important roles in society. The first documented women’s group amongst the Yoruba - *Egbe Alasalatu* - was founded in 1870 in Oṣogbo to organize religious activities amongst women, particularly women’s contributions to and participation in social events such as weddings and naming ceremonies and preparing women’s bodies for Islamic burial. After some initial resistance, it was recognized by Imams and women were given a dedicated space in mosques and the organization gained prominence within society. *Asalatu* women’s groups are now widespread across Nigeria (Badru and Sackey 2013). The recent rise of ‘charismatic’ Islam has also increased the influence of women’s groups in Islam in Nigeria. Spearheaded by *Nasirul-Lahi-L-Fatih* Society of Nigeria (NASFAT), the movement appropriates evangelical Christian forms/expressions (including groups and associations) as it seeks to compete with Pentecostal Christianity which has ‘moved into pole position in the fiercely competitive religious market place’(Obadare 2016). It includes mostly literate, upwardly mobile, urban Muslims but has not reached the Plateau Fulani who are mostly adherents of Izala and JIBWIS. Of all the Islamic civil society groups present in Plateau State, only JIBWIS has no women’s arm (Moddibbo 2012, Obadare 2016, Higazi 2013).

The Federation of Muslim Women’s Associations in Nigeria (FOMWAN) founded in 1985 is an umbrella organization that links Islamic women’s groups in Nigeria. FOMWAN has nationwide membership and is affiliated with over 500 national and international organizations. FOMWAN spreads Islam through *da’wah* (evangelism, proselytism) and advocates for Muslim girls’ education as a means to eradicating poverty and improving the status of Muslim women and children. It holds programmes to increase the retention rate of girls in school, continue education for married women and integrate literacy and vocational training into established Qur’anic schools (Uthman 2009). Nigeria has a strong tradition of Islamic women’s groups with similar goals, scope and impact to the Christian groups amongst the Ron, but Fulani women in this study are removed from this support through their inability to participate in public religious life and therefore have no access to the benefits of this collective action.

## Conclusions

The case studies presented here argue for greater understanding of the impact on empowerment and agency that can be made by collective action groups and demonstrate the prominence of culture and religion and social stratification determining the lives of women. Culture and religion emerged as important factors for socio-economic growth. Gender analysis provided sensitive information on both the opportunities and limitations of SHG equity and efficiencies.

Religious reform and the pluralistic nature of rural villages on the Jos Plateau restrict movement of Fulani women and account for the lack of Fulani women’s self-help groups. This lack of Fulani women’s groups results in the failure to recognize the potentials of women as a group. The non-participation of Fulani women on the Plateau in decision-making activities and limited or lack of any social interaction with their ‘indigenes’ counterparts could result in gender unequal sharing in costs and benefits relevant to the agency of women (Agarwal, 2000).

By contrast, the homogeneous Fulani community of the KGR with limited interaction from government, institutions and policies tended to encourage women’s self-help groups to adopt collective action in providing and managing access to resources. Unrestricted mobility enabled livelihood diversification to cope with stresses and challenges, increasing the empowerment and agency of Fulani women despite the cultural, social, religious and gender biases that limit women groups’ ability and strength. These biases continue to inhibit the women self-help groups, frustrating their efforts and denying them participation and involvement in some service delivery interventions. Reinforcing actions that universally support the activities of women’s self-help groups in the form of institutional support would be required to start and sustain transformative change.
